# Placental mitochondrial DNA content and placental abruption: a pilot study

**DOI:** 10.1186/s13104-015-1340-4

**Published:** 2015-09-16

**Authors:** Chunfang Qiu, Sixto E. Sanchez, Karin Hevner, Daniel A. Enquobahrie, Michelle A. Williams

**Affiliations:** Center for Perinatal Studies, Swedish Medical Center, Seattle, WA USA; Sección de Post Grado, Facultad de Medicina Humana, Universidad San Martín de Porres, Lima, Peru; A.C. PROESA, Lima, Peru; Department of Epidemiology, School of Public Health, University of Washington, Seattle, WA USA; Department of Epidemiology, Harvard School of Public Health, Boston, MA USA

**Keywords:** Placental abruption, Mitochondrial dysfunction, Placental mitochondrial DNA, Pregnancy, Biomarkers

## Abstract

**Background:**

Mitochondrial biogenesis and adequate energy production are important for embryogenesis and placentation. Previous studies documented alterations in maternal blood mitochondrial DNA (mtDNA) copy number—a marker of mitochondrial dysfunction—in pregnancies complicated by placental abruption. To further understand the role of mitochondrial dysfunction in the pathogenesis of placental abruption, we conducted a pilot study using placental specimen collected from 103 placental abruption cases and 102 non-abruption controls. Real-time quantitative polymerase chain reaction (PCR) was used to assess the relative copy number of mtDNA in DNA extracted from placental samples collected immediately after delivery. Logistic regression procedures were used to estimate adjusted odds ratios (OR) and 95 % confidence intervals (CI).

**Results:**

Higher odds of placental abruption was observed with increasing mtDNA copy number (*p* value for trend = 0.05). The odds of placental abruption was elevated among women who delivered placentas with higher mtDNA copy number (≥120.5, the median) as compared with those with lower values (<120.5) (adjusted OR = 2.38; 95 % CI 1.11–5.08).

**Conclusion:**

We found preliminary evidence for associations of target tissue-specific mitochondrial dysfunction with an adverse perinatal outcome, placental abruption. Larger studies and replication of findings in other populations will further our understanding of relationships between cellular and genomic biomarkers of normal and abnormal placental function and vascular placental disorders.

## Background

Placental abruption, the premature separation of the placenta from the uterus, is a life threatening obstetrical condition that complicates approximately 1 % of all pregnancies [1–3]. The condition occurs in much higher frequencies among women with multi-fetal gestation, coagulopathies, acquired forms of thrombophilia, uterine anomalies, abdominal trauma, hypertension, premature rupture of membranes, maternal-fetal hemorrhage, and intrauterine infections [4–8]. Advanced maternal age, grand-multiparity, and maternal cigarette smoking have been identified as placental abruption risk factors [6–8]. Pathophysiologic mechanisms involved in placental abruption and related perinatal disorders (e.g., preterm birth, preeclampsia, and intrauterine growth restriction) include uteroplacental ischemia, under perfusion, chronic hypoxia, and infarctions.

Alterations in mitochondrial DNA (mtDNA) density (copy number) in various tissues, including whole blood, has emerged as a possible biomarker of mitochondrial dysfunction, a risk factor for diverse cardiometabolic and neurodegenerative disorders, cancers [9–11], and adverse perinatal outcomes [12]. For example, we recently reported associations of alterations in mtDNA copy number in maternal blood with the risk of placental abruption [12]. In view of this emerging literature, we postulated an association between mtDNA copy number in placenta with risk of placental abruption. We used data and placental tissues remaining from an earlier study [13] to preliminarily test our hypothesis.

## Results and discussion

Socio-demographic, medical, and reproductive characteristics of placental abruption cases and controls are summarized in Table 1. Median values of placental mtDNA copy number were higher among placental abruption cases as compared with controls (144.3 vs. 120.5; p-value = 0.14), though the difference did not reach statistical significance. After controlling for confounding by maternal pre-pregnancy body-mass index, employment status during pregnancy, and gestational age at delivery we noted a trend of increased risk of placental abruption with increased mtDNA copy number (p for trend test = 0.05). The fully adjusted odds ratios (ORs) of placental abruption for the successive quartiles of mtDNA copy number, compared with the referent (first quartile) were 0.72 [95 % confidence interval (95 % CI) 0.22–2.35], 1.91 (95 % CI 0.66–5.52) and 2.15 (95 % CI 0.74–6.21) (p for trend = 0.05) (Table 2). Because the odds of placental abruption appeared to be increased only in the upper two quartiles, we repeated analyses after creating a dichotomous variable for high (upper 2 quartiles) versus low (lower 2 quartiles) mtDNA copy number using the median (120.5). From these analyses, we noted that the odds of placental abruption were 2.38-fold higher among women with higher (≥120.5) placental mtDNA copy number as compared with those with lower (<120.5) values (adjusted OR = 2.38; 95 % CI 1.11–5.08).

**Table 1:** Maternal characteristics of the placental abruption cases and controls

Characteristics	Study groups				P-value^b^
	Placental abruption (N = 103)		Comparison group (N = 102)		
	n	%	n	%	
Maternal age at delivery (years)^a^	27.0 ± 6.6		26.8 ± 5.8		0.89
Maternal age at delivery (years)					
<20	18	17.5	10	9.8	0.29
20–34	71	68.9	78	76.5	
≥35	14	13.6	14	13.7	
Gravidity					
1	45	43.7	37	36.3	0.21
2–3	40	38.8	52	51.0	
≥4	18	17.5	13	12.7	
Nulliparous	46	44.7	37	36.3	0.22
Maternal education ≤ high school	76	73.8	69	67.7	0.29
Single marital status	19	18.5	14	13.7	0.29
Employed during pregnancy	58	56.3	44	43.1	0.06
Received prenatal care	94	91.3	97	95.1	0.41
Did not take prenatal vitamins	11	10.7	14	13.7	0.53
Smoked during pregnancy	3	2.9	1	1.0	0.62
Alcohol consumption during pregnancy	9	8.7	0	0.0	0.003
Drug abuse during pregnancy	1	1.0	0	0.0	–
Chronic hypertension	3	2.9	1	1.0	0.42
Preeclampsia	11	10.7	4	3.9	0.11
Pre-pregnancy body mass index (kg/m^2^)^a^	23.5 ± 3.2		23.6 ± 3.5		0.84
Lean (<18.5)	4	3.9	1	1.0	0.57
Normal (18.5–24.9)	75	72.8	74	72.5	
Overweight (25–29.9)	18	17.5	21	20.6	
Obese (≥30.0)	6	5.8	6	5.9	
Gestational age at delivery	34.7 ± 4.3		39.1 ± 1.4		<0.001
Infant birthweight (g)	2388 ± 921		2835 ± 939		<0.001
Stillbirth	21	20.4	0	0.0	<0.001

^a^Mean ± standard deviation (SD)

^b^P-value from Student’s *t* test for continuous variables and Chi square test/Fisher’s Exact test for categorical variables

**Table 2:** Odds ratio (OR) and 95 % confidence interval (CI) for placental abruption in relation to categories of placental mitochondrial DNA copy number

Placental mitochondrial DNA copy number	Placental abruption cases (N = 103)	Controls (N = 102)	Adjusted OR^a^ (95 %CI)
Quartile 1 (<86.5)	22 (21.4)	26 (25.5)	1.00 (reference)
Quartile 2 (86.5–120.4)	17 (16.5)	25 (24.5)	0.72 (0.22–2.35)
Quartile 3 (120.5–191.4)	27 (26.2)	25 (24.5)	1.91 (0.66–5.52)
Quartile 4 (≥191.5)	37 (35.9)	26 (25.5)	2.15 (0.74–6.21)
P-value for linear trend			0.05
<Median (<120.5)	39 (37.9)	51 (50.0)	1.00 (reference)
≥Median (≥120.5)	64 (62.1)	51 (50.0)	2.38 (1.11–5.08)

^a^Adjusted for maternal pre-pregnancy body mass index, employed during pregnancy and gestational age at delivery

Higher placental mtDNA copy number is associated with higher risk of placental abruption. The odd of placental abruption was 2.38-fold higher among women who delivered placentas with mtDNA copy number at or above the median as compared with those women who delivered placentas with mtDNA copy number below the median. To the best of our knowledge, this is the first report that documents a positive association between placental mitochondrial density (copy number) and placental abruption. These preliminary data are in general agreement with earlier research findings from our group [12]. We examined the association of maternal whole blood mtDNA copy number with the odds of placental abruption [12] and found that the odds of placental abruption was positively associated with maternal blood mtDNA copy number. Specifically, we found a 1.6-fold increased odds of placental abruption for mothers with blood mtDNA copy number ≥336.9, as compared to those with values <336.9. Our present study further extends the literature, by documenting a positive association of placental abruption risk with evidence of placental mitochondrial dysfunction as reflected by increased mtDNA content/copy number.

The placenta, a highly complex organ which serves as the site for nutrient, water, and waste exchange between the mother and her fetus, plays an important role in modulating placental function and fetal growth. Further, as an immune-endocrine organ that constitutes the intrauterine environment, the placenta plays an important role in mediating the impact of environmental and metabolic insults on fetal growth. Mitochondria, cellular structures that provide most of the energy production in cells, control a number of important cellular processes, (e.g., fat metabolism, steroid synthesis, and apoptosis) and are likely to play a central role in placental implantation, growth and development.

Several lines of evidence have provided plausible biochemical mechanisms for the observed association of placental mitochondrial dysfunction with placental abruption. First, mtDNA is known to be highly susceptible to oxidative damage. This vulnerability has been attributed to absence of histones or DNA-binding proteins, limited DNA repair mechanisms, intron-less mitochondrial genes, rapid replication, and insufficient and inaccurate proofreading system [14–16]. Mitochondrial DNA damage, particularly deletions in the control regions of the circular mitochondrial genome, as reflected by alterations in mtDNA copy number, has also been shown to alter mitochondrial gene expression and lead to a deficiency in oxidative phosphorylation and enhanced generation of ATP by glycolysis [17]. Second, oxidative stress may contribute to alterations in mitochondrial function and increased mtDNA copy numbers through mechanisms that involve reactive oxygen species (ROS)-induced damage to cellular structural elements (including the lipid membranes of mitochondria) [18]. ROS may also affect mitochondrial function by damaging DNA and impairing electron chain transport, which initiates a compensatory response that increases mtDNA copy number [17]. Evidence for this hypothesized mechanism is supported by findings from experimental animal studies documenting increased mitochondrial damage and mtDNA copy numbers with increasing exposure to pro-oxidants [18]. Taken together, available epidemiological and other data underscore biologically plausible mechanisms that may account for the association observed in our preliminary study.

Several limitations of the present pilot study merit consideration. First, given the retrospective study design, we cannot establish the temporal relationship between elevated placental mtDNA copy numbers and the onset of placental abruption. Second, differential misclassification of maternal mtDNA copy number is a possibility, though unlikely, as all laboratory analyses were conducted without knowledge of participants’ case–control status. Third, although we required that all physician diagnosed placental abruption cases had documented evidence blood clot behind the placental margin accompanied by at least two of the following clinical signs and symptom (vaginal bleeding late pregnant not associate with placenta previa or cervical lesions; uterine tenderness and/or abdominal pain; and poor fetal tracings suggestive of fetal distress or stillbirth), we cannot rule out the possibility that less severe placental abruption cases were missed classified as non-cases. Fourth, we took biopsy samples from four systematically selected sites from each placenta, and we pooled the tissues sampled from each participant to then estimate mtDNA content for the pooled sample. Hence, in this pilot and feasibility study, we are not able to report the coefficient of variation for mtDNA content according to different biopsies. Future studies are needed to assess mtDNA content according to specific cell types. Fifth, we used well-established commercially available primers, reagents and protocols for mtDNA content determination, with 2 housekeeping genes (BECN1 and NEB) for normalization (NovaQUANT™ Human Mitochondrial to Nuclear DNA Ratio Kit); hence we recognize that our quantitative real-time PCR experiments may not strictly adhere to the MIQE guidelines [19]. Lastly, although we controlled for multiple confounding factors, we cannot exclude the possibility that the odds ratios reported are unaffected by residual confounding.

## Conclusions

Our results suggest that mtDNA copy number in placental tissue, a target tissue, may be associated with placental abruption. Future research that includes larger study populations and replication efforts will be needed to confirm our preliminary observations and expand the scope of research to include assessment of other measures of mitochondrial function and uteroplacental metabolism. Finally, analyses that interrogate the influences of variation in the mitochondrial genome in maternal, fetal and uteroplacental compartments are also warranted as such studies will elucidate the molecular mechanisms of maternal, fetal and uteroplacental bioenergetics during pregnancy.

## Methods

Study subjects were selected from participants of a case–control study designed to investigate risk factors of placental abruption. Study population and data collection procedures, described before, were briefly as follows [13]. Participants were women who delivered at the Hospital Nacional dos de Mayo, Instituto Especializado Materno Perinatal, Hospital Edgardo Rebagliati Martins, Hospital Nacional Hipolito Unanue, and Hospital Nacional Docente Madre Niño San Bartolomè in Lima, Peru, during the period from September 2006 through September 2008. This study was approved by the Institutional Review Board of the University of Washington. All study participants provided written informed consent.

All participating hospitals are tertiary care reference hospitals that manage high-risk pregnancies in Lima, Peru; and experienced maternal-fetal-medicine specialists cared for all patients. The diagnosis of placental abruption was based on clinical examination performed by the attending physician. For the research diagnosis of placental abruption, we required evidence of blood clot behind the placenta accompanied by at least two of the following signs and symptoms: (1) vaginal bleeding in late pregnancy that was not associated with placenta previa or cervical lesions; (2) uterine tenderness and/or abdominal pain; and (3) fetal distress or death. Controls were selected from eligible women who delivered at the participating institutions during the study period. Eligible controls were women who did not have a diagnosis of placental abruption and whose medical record review later confirmed this fact. For this present pilot and feasibility study, we randomly selected 103 consecutive placental abruption cases from a total sample of 383 cases. Additionally, we sampled 102 non-abruption controls from a total of 369 controls.

Study subjects were recruited during their labor and delivery hospital stay. We used a standardized, structured questionnaire to collect information regarding maternal sociodemographic, medical, reproductive, and lifestyle characteristics during in-person interviews. All interviews were conducted by trained research interviewers. Information collected during the interviews included maternal age, marital status, employment status during pregnancy, medical history, pre-pregnancy weight, and smoking and alcohol consumption during pregnancy. Maternal and infant records were reviewed to collect detailed information concerning antepartum, labor, and delivery characteristics, as well as conditions of the newborn. Maternal anthropometric measures (e.g., height and weight) were taken at the time of the interview. Gestational age was based on the date of the last menstrual period and was confirmed by an ultrasound examination performed before 20 weeks gestation. Pre-pregnancy body mass index (BMI), a measure of overall maternal adiposity, was calculated as weight in kilograms divided by height in meters squared. Women were classified as lean (BMI <18.5 kg/m^2^), normal (BMI = 18.5–24.9 kg/m^2^), overweight (BMI = 25.0–29.9 kg/m^2^) and obese (BMI ≥30.0 kg/m^2^).

Placentas were collected immediately after delivery. Placentas were weighed, double bagged and transported in coolers. The chorionic plate and overlying membranes were removed, and tissue biopsies (approximately 0.5 cm^3^ each) were randomly obtained from 8 sites (4 maternal and 4 fetal). Care was taken to avoid the chorioamniotic membrane contamination. For this present study, biopsy samples taken from the fetal side, which consisted of trophoblast tissues, intervillous tissues, and chorionic villi, were sampled for genomic DNA extraction. Biopsies were placed in cryotubes, snap frozen in liquid nitrogen, and stored at −80 °C until analysis.

A pooled sample (~20 g) from 4 fetal placental samplings, representing the intervillous tissues, and chorionic villi, was homogenized using a Tissue Tearor (Biospec Products Inc., Bartlesville, OK, USA) in a lysis buffer from the Qiamp DNA Mini Kit (Qiagen Inc, Valencia, CA, USA) with added Proteinase Kt. DNA was extracted using a standardized protocol adapted from Qiamp DNA Mini Kit (Qiagen Inc., Valencia, CA, USA). DNA, from aliquots of placental biopsies, was extracted using standard salting-out procedures [20] in a core facility (Roswell Park Cancer Institute, Buffalo, NY, USA). For quantification of mtDNA copy number, real-time DNA PCR analysis was performed with the NovaQUANT™ Human Mitochondrial to Nuclear DNA Ratio Kit (catalog #72620 EMD Millipore, Billerica, MA, USA) according to the manufacturer’s instructions [21]. This kit provides plates pre-aliquoted with the primers to compare the levels of two nuclear genes (BECN1 and NEB) to two mitochondrial genes (ND1 and ND6) in a real-time quantitative polymerase chain reaction. Mitochondrial ND1 [GenBank KP875569 (3314–3467)] and ND6 [GenBank: KP875569 (14341–14436)] encode NADH dehydrogenase 1 or NADH dehydrogenase 6 which are core subunit of the mitochondrial membrane respiratory chain NADH dehydrogenase (Complex I) that is believed to belong to the minimal assembly required for catalysis. Complex I functions in the transfer of electrons from NADH to the respiratory chain in mitochondria. BECN1 encodes beclin 1, autophagy related and locates in chromosome 17q21 with 13 exons [GenBank: AC016889.28 (89304–80432)]. NEB encodes nebullin, a giant protein component of the cytoskeletal matrix that coexists with the thick and thin filaments within the sarcomeres of skeletal muscle. NEB is located in chromosome 2q22 with 183 exons [GenBank: NG_009382.2 (16079–16195)]. 2 ng of DNA was added to 80 μL of SYBR Select Master Mix (catalog #4472908 Life Technologies, Carlsbad, CA, USA) then 20 GenBank KP875569 (3314–3467L applied to each of the four wells of pre-aliquoted primers. Plates were run in the ABI Prism 7000 sequence detection system (Applied Biosystems, Foster City, CA, USA) with the cycling conditions: 95 °C for 10 min followed by 40 Cycles of: 95 °C for 15 s 64 °C for 1 min. Analysis was done using the ABI Prism 7000 SDS software RQ Study Application version 1.1. Relative copy number method was used to calculate the mtDNA copy number. This method results in the highest sample throughput. Ct values, where Ct is defined as the cycle number in which fluorescence first crosses the threshold, obtained for each of the two target genes and two reference genes are used once to determine mtDNA copy numbers. This is accomplished by averaging the copy numbers calculated from the ND1/BECN1 pair and the ND6/NEB pair. To calculate copy number, calculation N = 2^−ΔCt^ where ΔCt1 = Ct^ND1^ − Ct^BECN1^ and ΔCt2 = Ct^ND6^ − Ct^NEB^, then do the average of 2^−ΔCt1^ and 2^−ΔCt2^. All assays were performed without knowledge of pregnancy outcome.

We examined frequency distributions of maternal socio-demographic, medical characteristics, and medical and reproductive histories according to placental abruption case–control status. Continuous data were checked for normality and are presented as arithmetic mean ± standard deviation (SD) when normally distributed or median with interquartile range (IQR) when data were not normally distributed. Since the distribution of placental mtDNA copy number was skewed for cases and controls, differences in median values of mtDNA copy number between placental abruption cases and control groups were compared based on the Mann–Whitney *U* test. Categorical data are presented as frequencies (percent) and numbers. To estimate the relative association of placental abruption with placental mtDNA copy number, we categorized each subject according to quartiles determined by the distribution of mtDNA relative copy number among controls. We used the lowest quartile as the referent group, and we estimated odds ratios (OR) and 95 % confidence intervals (95 % CI) for each of the upper three quartiles. We then contrasted the highest two quartile with the lowest two quartiles combined since we observed no evidence of association of placental abruption with mtDNA across the lower two quartiles (below the median). We also evaluated linear trends in risk by considering the four quartiles as a continuous variable after assigning a score to each quartile [22]. To assess confounding, covariates were entered into a logistic regression model one at a time. We then compared the adjusted and unadjusted odds ratios [22]. Final logistic regression models included covariates that altered unadjusted odds ratios by at least 10 %, as well as those covariates of a priori interest (e.g., maternal pre-pregnancy BMI). We considered the following covariates as possible confounders in this analysis: maternal age, parity, smoking during pregnancy, no prenatal care, and gestational age at delivery. All analyses were performed using STATA 9.0 statistical software.

## Abbreviations

ATP: adenosine triphosphate
BECN1: beclin 1, autophagy related
BMI: pregnancy body mass index
CI: confidence interval
IQR: inter-quartile range
mtDNA: mitochondrial DNA
ND1: (mitochondrially encoded) NADH dehydrogenase 1
ND6: (mitochondrially encoded) NADH dehydrogenase 6
NEB: nebullin
OR: odds ratio
ROS: reactive oxygen species
SD: standard deviation
